# Identification of hub genes and pathways associated with cellular senescence in diabetic foot ulcers via comprehensive transcriptome analysis

**DOI:** 10.1111/jcmm.18043

**Published:** 2023-11-20

**Authors:** Yike Huang, Dongqing Wang, Wen Zhang, Xue Yuan, Ke Li, Yuanyuan Zhang, Mingqiang Zeng

**Affiliations:** ^1^ Department of Emergency The First Affiliated Hospital of Chengdu Medical College Chengdu China; ^2^ School of Clinical Medicine, Chengdu Medical College Chengdu China; ^3^ Department of Medical Laboratory Xindu District People’ s Hospital of Chengdu Chengdu China; ^4^ Department of Pediatrics Chongqing Bishan Area Women and Children Hospital Chongqing China

**Keywords:** cellular senescence, diabetic foot ulcers, differentially expressed genes, hub genes, potential target

## Abstract

This research aimed to find important genes and pathways related to cellular senescence (CS) in diabetic foot ulcers (DFU) and to estimate the possible pathways through which CS affects diabetic foot healing. The GSE80178 dataset was acquired from the Gene Expression Omnibus (GEO) database, containing six DFU and three diabetic foot skin (DFS) samples. The limma package was used to identify differentially expressed genes (DEGs). At the same time, DEGs associated with CS (CS‐DEGs) were found using the CellAge database. Gene Ontology (GO) and Kyoto Encyclopedia of Genes and Genomes (KEGG) enrichment analyses were conducted on the CS‐DEGs. A protein–protein interaction (PPI) network was built using the String database, and the cytoHubba plug‐in within Cytoscape helped identify hub genes. Lastly, the miRNA‐TF‐mRNA regulatory network for these hub genes was established. In total, 66 CS‐DEGs were obtained. These genes mainly focus on CS, Kaposi sarcoma‐associated herpesvirus infection and Toll‐like receptor signalling pathway. Eight hub genes were identified to regulate cell senescence in DFU, including TP53, SRC, SIRT1, CCND1, EZH2, CXCL8, AR and CDK4. According to miRNA‐TF‐mRNA regulatory network, hsa‐mir‐132‐3p/SIRT1/EZH2 axis is involved in senescence cell accumulation in DFU.

## 1 INTRODUCTION

With the worldwide increase in diabetes cases, the occurrence of diabetic foot complications is also on the rise. According to current statistics, about 80% of patients with lower limb amputation will have diabetic foot before surgery.^1^ The pathological mechanisms behind diabetic foot complications involve endogenous factors such as alterations in nerves, blood vessels, immunity, metabolism and chronic infection, along with excessive inflammation, unresponsiveness of epidermal or dermal cells to repair signals. Additionally, exogenous factors, such as biofilm formation due to drug‐resistant microorganisms, also contribute to the condition.^2^ Such a high disability rate and high mortality not only cause a decline in the quality of life of patients, but also lead to a heavy medical and nursing burden, so it is urgent to find new treatment options.

Growing evidence indicates that cellular senescence (CS) is closely linked to the onset and progression of diabetic foot wound healing.^3^ CS is a state of permanent cell cycle arrest accompanied by a decline in function. The primary cause of CS is telomere shortening or stress‐induced senescence. Senescent cells release a substantial amount of proinflammatory cytokines and chemokines, a phenomenon known as the senescence‐associated secretory phenotype (SASP), which can harm pancreatic β‐cells.^4^ Senescent cells play complex roles in type II diabetes pathophysiology by participating in adipose tissue dysfunction, directly affecting pancreatic β‐cell function, and promoting SASP‐mediated tissue damage.^5^, ^6^ Many metabolic processes in diabetes can also stimulate the formation of senescent cells, such as altered lipid metabolism and high circulating glucose.^7^ Thus, senescent cells may be a major cause and consequence of tissue damage and abnormal metabolic changes, central to the pathogenic circuit of diabetes.^8^ The diabetic microenvironment also promotes CS.^9^ Recent studies have shown that hyperinsulinemia leads to a premature senescent transcriptomic and secretory profile in mature adipocytes,^10^ while also causing insulin resistance in neurons, which results in a senescence‐like phenotype.^11^ Additionally, recent research indicates that sustained hyperinsulinemia contributes to the senescence of hepatocytes.^12^ CS plays a crucial role in the efficient healing of acute wounds early on, followed by clearance of senescent cells by macrophage‐dominated immune surveillance. On the contrary, senescent cells play a negative role in the healing process of chronic wounds, and the chemotaxis of macrophages is significantly reduced in chronic wounds, resulting in impaired ability to migrate and clear the sites of senescent cell accumulation (such as senescence‐associated secretory expression phenotype factor responses), leading to aggregation of senescent cells.^3^, ^13^ Senescent cells release a variety of SASPs that influence the surrounding microenvironment, indirectly and directly impacting the healing and regeneration process, which includes factors such as cell plasticity, angiogenesis and matrix remodelling. More and more evidence show that blocking CS or clearing senescent cells can promote the healing of chronic wounds, and CS is expected to be a therapeutic target for chronic wounds.^14^, ^15^, ^16^ In conclusion, preventing CS or eliminating senescent cells is anticipated to be a new therapeutic strategy for diabetic foot ulcers (DFU).

In the era of genomics, gene chips have become a popular tool for investigating the pathogenesis of diseases, offering novel insights into the development of DFU at the genetic level.^17^, ^18^ Thus, this study seeks to examine the connection between DFU and CS through bioinformatics analysis, aiming to offer valuable insights for the further treatment of DFU.

## 2 MATERIALS AND METHODS

### 2.1 Raw data collection

The gene expression profile of GSE80178 (https://www.ncbi.nlm.nih.gov/geo/query/acc.cgi?acc=GSE80178) was obtained from the Gene Expression Omnibus (GEO) database,^19^ a public repository hosting numerous high‐throughput sequencing and microarray data sets contributed by research institutions globally. The GSE80178 dataset^20^ was built on the Affymetrix GPL16686 platform (Affymetrix Human Gene 2.0 ST Array) and includes six DFU and three DFS samples.

### 2.2 Data preprocessing and integration

The limma package (version: 3.40.2) in R software was utilized to analyse the differential expression of mRNAs. The *p*‐value was examined to adjust for false positive outcomes in GEO datasets. ‘*p* < 0.05 and |logFC| ≥ 0.5’ were defined as the thresholds for the screening of differential expression of mRNAs. Probe sets without corresponding gene symbols were removed, and genes with multiple probe sets were averaged. We acquired 279 CS‐related genes from the CellAge database (https://genomics.senescence.info/cells/), a resource for CS‐related genes established from genetic manipulation experiments in various human cell types. The intersection of genes from the CellAge database and differentially expressed genes (DEGs) in GSE80178 were considered as CS‐DEGs for DFU.

### 2.3 Gene Set Enrichment Analysis

To gain a deeper understanding of the biological processes linked to DFU, we conducted Gene Set Enrichment Analysis (GSEA) using the clusterProfiler package.^21^ Hallmark and Canonical Pathways gene sets were obtained from the MSigDB collections (https://www.gsea‐msigdb.org/gsea/msigdb/index.jsp). Adjusted *p*‐value <0.05 and FDR (qvalue) < 0.25 were regarded as the cut‐off criteria.

### 2.4 Enrichment analyses of DEGs

To gain a deeper understanding of the primary biological functions of CS‐DEGs, we employed the clusterProfiler package for analysing the Gene Ontology (GO) and Kyoto Encyclopedia of Genes and Genomes (KEGG) pathways that regulate CS‐DEGs both up and down. An adjusted *p*‐value <0.05 was deemed significant.

### 2.5 PPI network construction and module analysis

Search Tool for the Retrieval of Interacting Genes (STRING; http://string‐db.org) (version 11.5)^22^ can search for the relationship between the proteins of interest, such as direct binding relationships, or coexisting upstream and downstream regulatory pathways, to construct a protein–protein interaction (PPI) network with complex regulatory relationships. Interactions with a combined score above 0.4 were deemed statistically significant. Cytoscape (http://www.cytoscape.org) (version 3.9.0)^23^ was employed to visualize this PPI network. The molecular complex detection technology (MCODE) plug‐in for Cytoscape was utilized to analyse key functional modules, with selection criteria set as: K‐core = 2, degree cut‐off = 2, max depth = 100, and node score cut‐off = 0.2. Subsequently, KEGG and GO analyses of the involved modular genes were conducted using the clusterProfiler package.

### 2.6 Selection and analysis of core CS‐DEGs

The core CS‐DEGs were detected by applying the cytoHubba plug‐in within Cytoscape. Here, we used six common algorithms (MCC, MNC, Degree, Closeness, Radiality and EPC) to evaluate and select core CS‐DEGs. Next, we established a co‐expression network of these hub genes using GeneMANIA (http://www.genemania.org/),^24^ a dependable tool for detecting internal associations within gene sets.

### 2.7 MiRNA‐TF‐mRNA regulatory network analysis

To gain a deeper understanding of the regulatory mechanism of hub genes, we obtained TF‐target interactions through the Transcriptional Regulatory Relationships Unravelled by Sentence‐based Text mining (TRRUST).^25^ TRRUST is a database for predicting transcriptional regulatory networks, which includes target genes linked to TFs and the regulatory relationships between TFs. Furthermore, miRNA‐target interactions were obtained through miRWalk, a publicly available database that focuses on miRNA‐target interactions.^26^ In addition, GSE84971^27^ was downloaded from the GEO database, including miRNA expression profiling of primary fibroblast derived from DFU. ‘*p* < 0.05 and |logFC| ≥ 1’ were defined as the thresholds for the screening of differential expression of miRNAs. Significant miRNA of DFU were considered to be the intersections of miRNA from the miRWalk database and miRNA in GSE84971 (https://www.ncbi.nlm.nih.gov/geo/query/acc.cgi?acc=GSE84971). Lastly, miRNA‐target interactions and TF‐target interactions were combined to construct the miRNA‐TF‐mRNA regulatory network using Cytoscape.

### 2.8 RT‐qPCR analysis

In this study, 8 DFU samples and six non‐diabetic foot skin were used. Among them, patients in DFU group included four males and four females, with an average age of 68 years (55–73 years); The normal control group consisted of 3 males and 3 females with an average age of 56 years (from 45 to 65 years). The Ethics Committee of the First Affiliated Hospital of Chengdu Medical College approved this study, and all patients informed and agreed to participate. Total RNA was extracted from the sample with the TRIzol reagent (Thermo FisherSci, Inc.) and reverse transcribed into cDNA. The following thermal cycling conditions were adopted: melting at 95°C for 30 s, annealing at 95°C for 3 s and extending at 60°C for 30 seconds. Taking GAPDH as endogenous control, the results were calculated by 2^−ΔΔCt^ method. The primers used in this study are as follows: SIRT1: F, AAGTTGACTGTGAAGCTGTACG, R, TGCTACTGGTCTTACTTTGAGGG; EZH2: F, GGACCACAGTGTTACCAGCAT, R, GTGGGGTCTTTATCCGCTCAG; hsa‐mir‐132‐3p: F, AACCGGTTTGCTGGTGAA, R, GTGCAGGGTCCGAGGT.

### 2.9 Statistical analysis

All statistical analysis in this paper was performed using the Xiantao Scholarship platform (www.xiantao.love), an online bioinformatics analysis tool that was developed based on the R language.

## 3 RESULTS

### 3.1 Identification of DEGs

**FIGURE 1:**
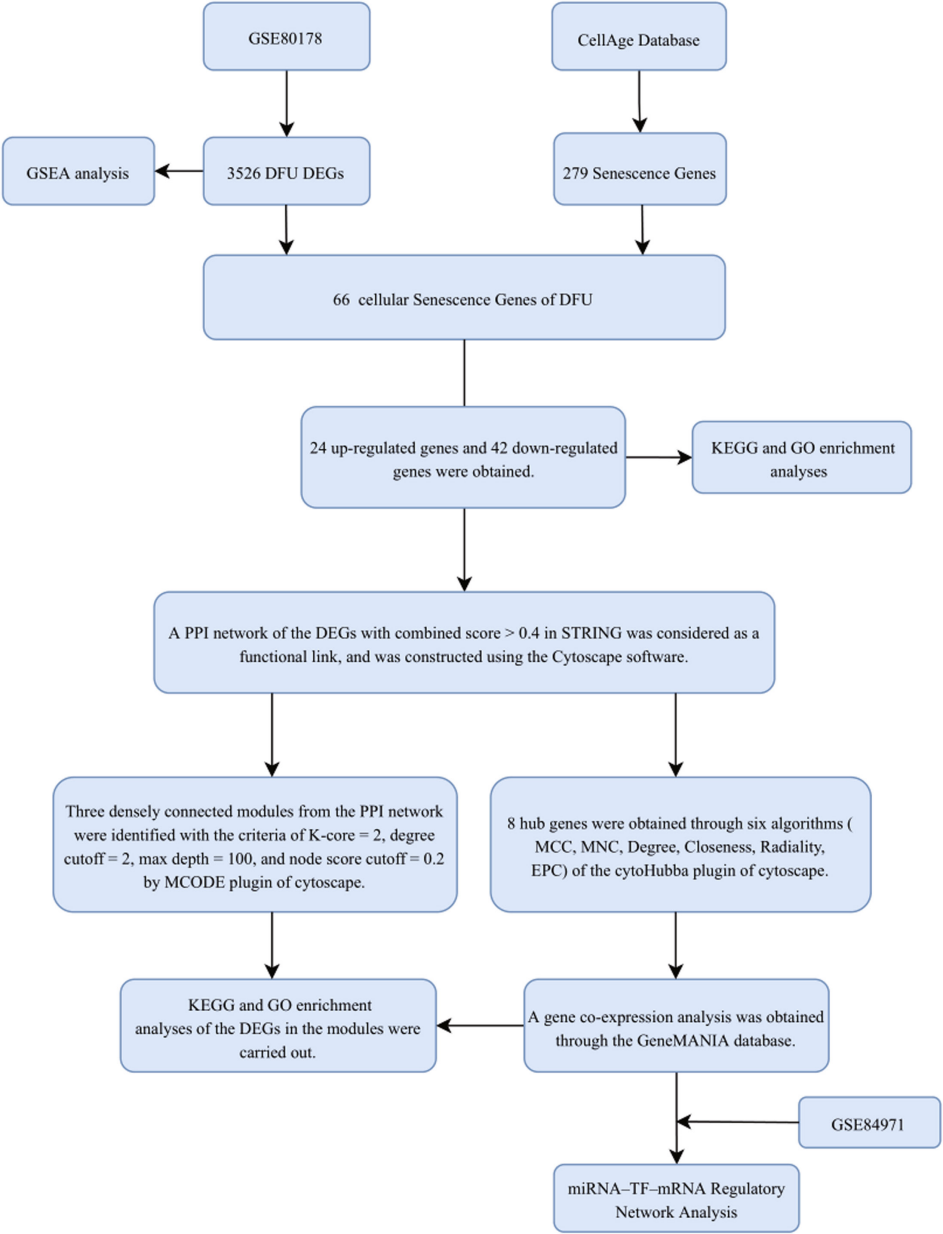
Research design flow chart.

**FIGURE 2:**
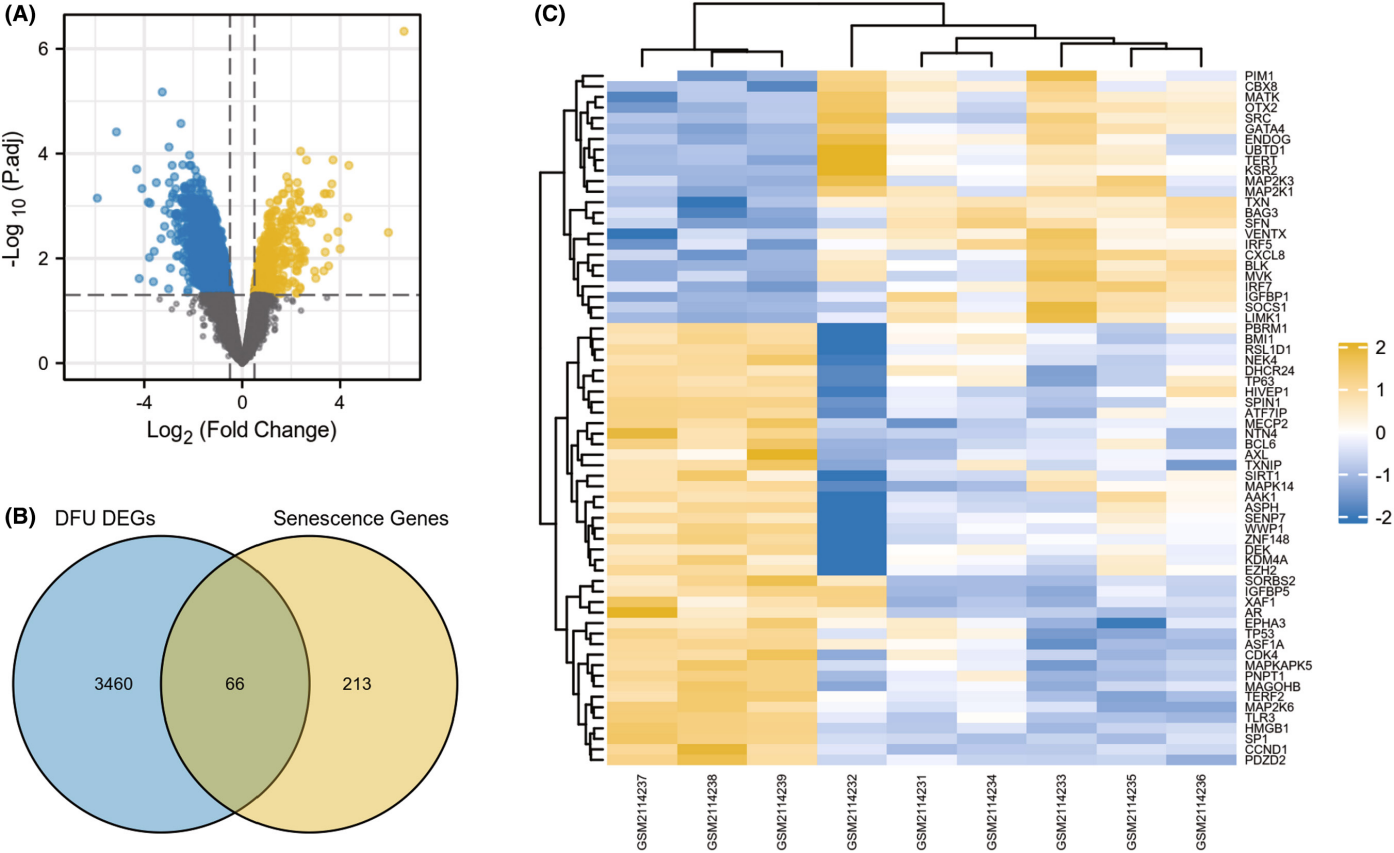
(A) The volcano map of GSE80178. (B) Venn diagram show that 66 overlapping cellular senescence‐differentially expressed genes (CS‐DEGs) in GSE80178 and CellAge database. (C) Heat map of overlapping CS‐DEGs. Upregulated genes are marked in light yellow; downregulated genes are marked in light blue.

The research flowchart for this study is presented in Figure 1. After normalizing the microarray data, 3526 DEGs were detected in GSE80178 (Figure 2A). Through Venn diagram analysis, we identified 66 overlapping CS‐DEGs in both GSE80178 and the CellAge database (Figure 2B). The expression heatmap of these 66 CS‐DEGs could effectively distinguish between DFU and DFS (Figure 2C). Table S1 provides detailed information on these overlapping CS‐DEGs.

### 3.2 Analysis of the functional characteristics

**FIGURE 3:**
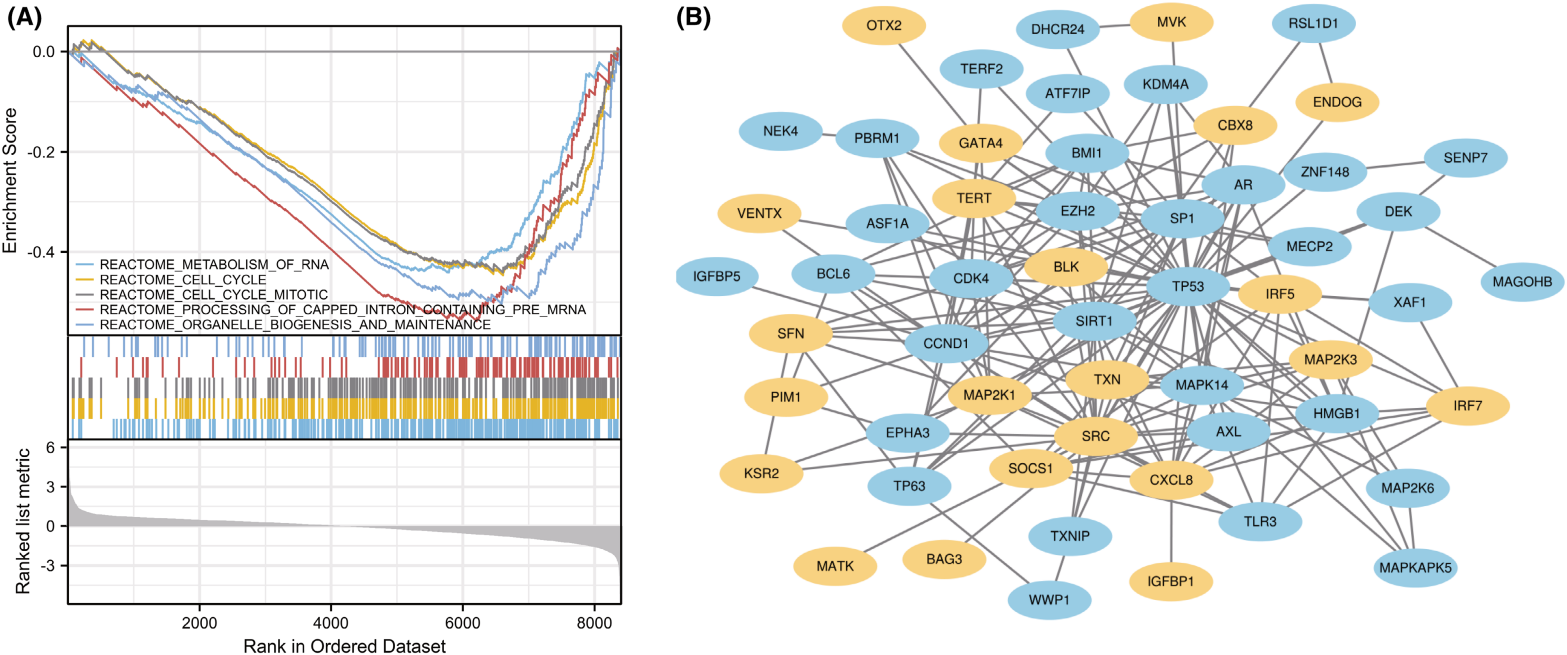
(A) The top five items enriched by GSEA. (B) Protein–protein interaction (PPI) network constructed using the STRING database. Upregulated genes are marked in light yellow; downregulated genes are marked in light blue.

**FIGURE 4:**
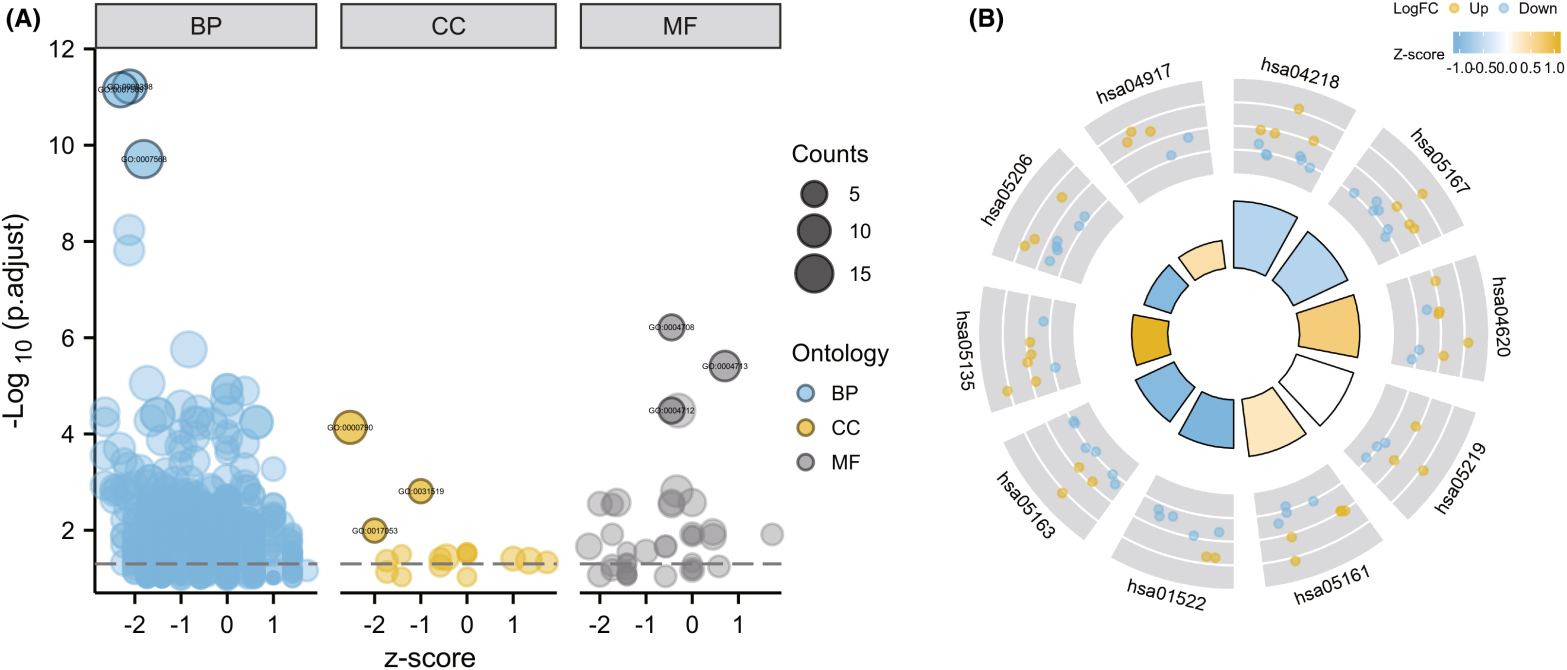
(A) Enrichment result of overlapping cellular senescence‐differentially expressed genes (CS‐DEGs) Gene Ontology (GO) term. The items above the dotted line are significant; (B) Enrichment result of overlapping CS‐DEGs KEGG pathway. Adjusted *p*‐value <0.05 was considered significant.

The top five enriched items identified by GSEA were RNA metabolism, cell cycle, mitotic cell cycle, processing of capped intron‐containing pre‐mRNA, and organelle biogenesis and maintenance (Figure 3A). To analyse the biological functions and pathways involved in CS‐DEGs, GO and KEGG pathway enrichment analyses were performed. The GO analysis results revealed that CS‐DEGs were significantly enriched in CS, cell aging, and aging in terms of biological processes (BP). Concerning cellular components (CC), the DEGs were mainly linked to nuclear chromatin, PcG protein complex, and transcriptional repressor complex. For molecular function (MF), DEGs were predominantly associated with MAP kinase kinase activity, protein tyrosine kinase activity and protein serine/threonine/tyrosine kinase activity (Figure 4A and Table 1). KEGG pathway analysis revealed that the DEGs were mainly involved in CS, Kaposi sarcoma‐associated herpesvirus infection, and the Toll‐like receptor signalling pathway (Figure 4B and Table 1).

**TABLE 1:** GO and KEGG enrichment analysis of overlapping CS‐DEGs.

Term	ID	Description	GeneRatio	*p*.adjust
BP	GO:0090398	Cellular senescence	11/65	6.06e–12
	GO:0007569	Cell aging	12/65	7.05e–12
	GO:0007568	Aging	15/65	1.95e–10
	GO:2000772	Regulation of cellular senescence	8/65	5.75e–09
	GO:0090342	Regulation of cell aging	8/65	1.53e–08
CC	GO:0000790	Nuclear chromatin	10/65	7.29e–05
	GO:0031519	PcG protein complex	4/65	0.002
	GO:0017053	Transcriptional repressor complex	4/65	0.010
	GO:0000782	Telomere cap complex	2/65	0.029
	GO:0000783	Nuclear telomere cap complex	2/65	0.029
MF	GO:0004708	MAP kinase kinase activity	5/65	6.10e–07
	GO:0004713	Protein tyrosine kinase activity	8/65	3.87e–06
	GO:0004712	Protein serine/threonine/tyrosine kinase activity	5/65	3.26e–05
	GO:0004674	Protein serine/threonine kinase activity	11/65	3.26e–05
	GO:0001228	DNA‐binding transcription activator activity, RNA polymerase II‐specific	9/65	0.002
KEGG	hsa04218	Cellular senescence	10/42	7.88e–07
	hsa05167	Kaposi sarcoma‐associated herpesvirus infection	10/42	2.25e–06
	hsa04620	Toll‐like receptor signalling pathway	8/42	2.25e‐06
	hsa05219	Bladder cancer	6/42	2.25e–06
	hsa05161	Hepatitis B	9/42	3.58e–06

Abbreviations: BP, biological processes; CC, cellular components; CS‐DEGs, cellular senescence‐differentially expressed genes; GO, Gene Ontology; KEGG, Kyoto Encyclopedia of Genes and Genomes; MF, molecular function.

### 3.3 PPI network construction and module analysis

**FIGURE 5:**
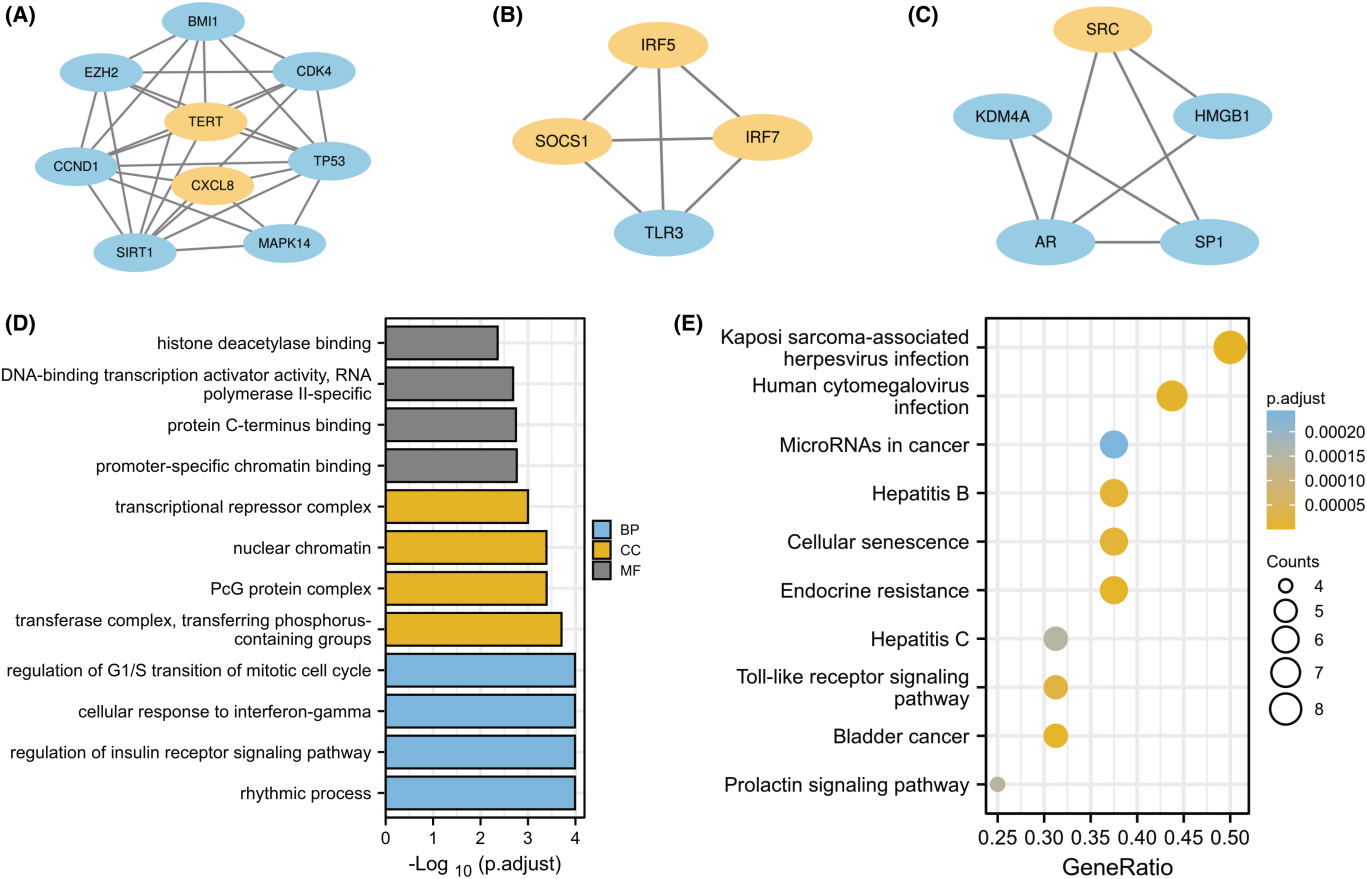
(A–C) Three significant gene clustering modules. (D, E) Gene Ontology (GO) and KEGG enrichment analysis of the modular genes.

The PPI network of CS‐DEGs with combined scores above 0.4 was created using Cytoscape, consisting of 58 nodes and 193 interaction pairs (Figure 3B). MCODE plug‐in of Cytoscape revealed three closely connected gene modules that comprised 18 CS‐DEGs and 41 interaction pairs (Figure 5A–C). GO analysis demonstrated that these genes are involved in rhythmic processes, regulation of insulin receptor signalling pathway, transferase complex, transferring phosphorus‐containing groups, PcG protein complex, promoter‐specific chromatin binding, and protein C‐terminus binding (Figure 5D). KEGG pathway analysis found that these genes are mainly associated with Kaposi sarcoma‐associated herpesvirus infection, bladder cancer, endocrine resistance, human cytomegalovirus infection, and CS (Figure 5E).

### 3.4 Selection and analysis of core CS‐DEGs

**FIGURE 6:**
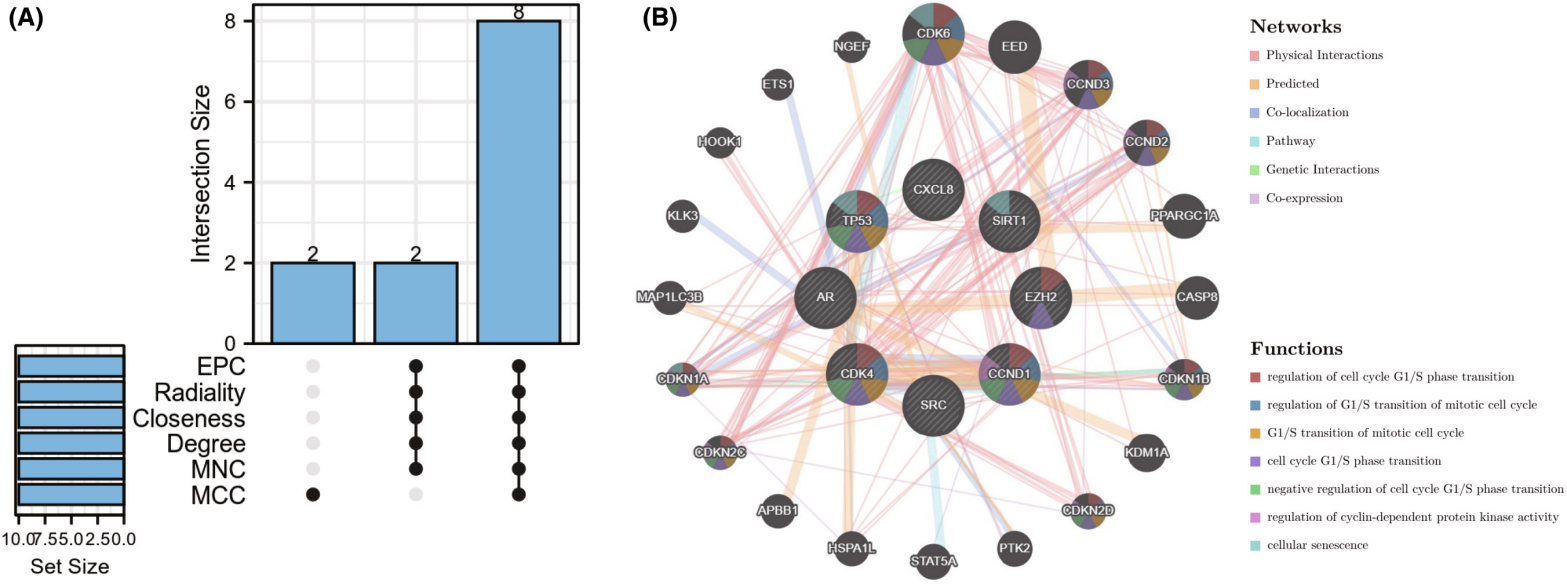
(A) The Venn diagram showed that six algorithms have screened out eight overlapping cellular senescence‐differentially expressed genes (CS‐DEGs). (B) Core CS‐DEGs and their co‐expression genes were analysed via GeneMANIA.

**FIGURE 7:**
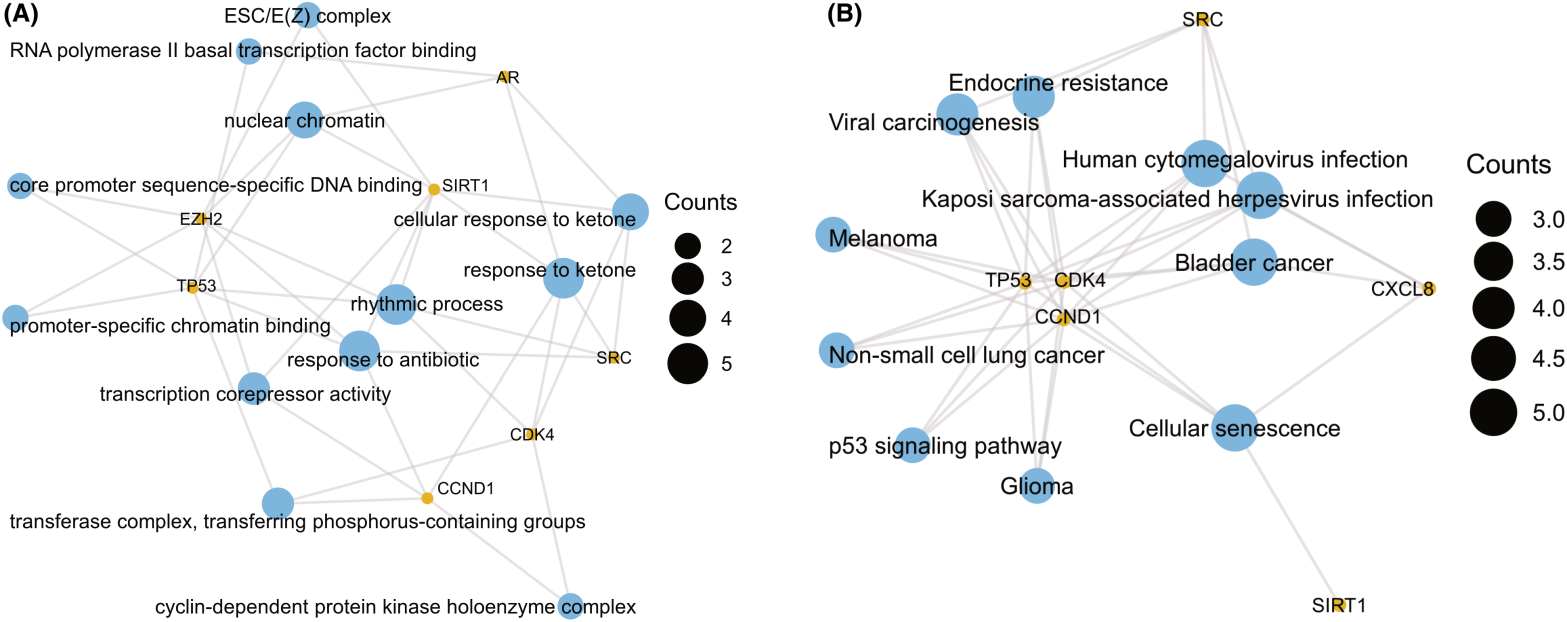
(A, B) Gene Ontology (GO) and KEGG enrichment analysis of core cellular senescence‐differentially expressed genes (CS‐DEGs). Adjusted *p*‐value <0.05 was considered significant.

Using the cytoHubba plug‐in's six algorithms, we calculated the top 10 hub genes (Table 2), and 8 overlapping hub genes were obtained through Venn diagram analysis, including TP53, SRC, SIRT1, CCND1, EZH2, CXCL8, AR, and CDK4 (Figure 6A). Table 3 shows their full names and related functions. Using the GeneMANIA database, we analysed the co‐expression network and related functions of these genes. These genes showed a complex PPI network with physical interactions of 44.88%, predicted interactions of 19.39%, co‐localization of 12.51%, pathway of 11.63%, genetic interactions of 6.28%, and co‐expression of 5.32% (Figure 6B). These genes are related to the regulation of cell cycle G1/S phase transition, regulation of G1/S transition of mitotic cell cycle, and G1/S transition of the mitotic cell cycle (Figure 6B). Similarly, GO analysis showed that these genes are related to response to ketone, cellular response to ketone, nuclear chromatin, ESC/E(Z) complex, RNA polymerase II basal transcription factor (TF) binding and transcription corepressor activity (Figure 7A). KEGG pathway analysis found that these genes are mainly associated with bladder cancer, CS, and Kaposi sarcoma‐associated herpesvirus infection (Figure 7B).

**TABLE 2:** The top 10 CS‐DEGs rank in cytoHubba.

MCC	MNC	Degree	Closeness	Radiality	EPC
TP53	TP53	TP53	TP53	TP53	TP53
CCND1	SRC	SRC	SRC	CCND1	CCND1
SIRT1	SIRT1	CCND1	CCND1	SRC	SIRT1
CDK4	CCND1	SIRT1	SIRT1	SIRT1	SRC
EZH2	MAPK14	SP1	MAPK14	SP1	CXCL8
SRC	EZH2	MAPK14	SP1	MAPK14	MAPK14
AR	CXCL8	CXCL8	CXCL8	EZH2	SP1
TERT	SP1	EZH2	EZH2	CXCL8	AR
BMI1	AR	AR	AR	AR	EZH2
CXCL8	CDK4	CDK4	CDK4	CDK4	CDK4

Abbreviation: CS‐DEGs, cellular senescence‐differentially expressed genes.

**TABLE 3:** The details of the core CS‐DEGs.

No.	Gene symbol	Full name	Function
1	TP53	Tumour protein P53	The encoded protein responds to diverse cellular stresses to regulate expression of target genes, thereby inducing cell cycle arrest, apoptosis, senescence, DNA repair, or changes in metabolism
2	SRC	SRC proto‐oncogene	This proto‐oncogene may play a role in the regulation of embryonic development and cell growth
3	SIRT1	Sirtuin 1	This gene encodes a member of the sirtuin family of proteins, homologues to the yeast Sir2 protein. Members of the sirtuin family are characterized by a sirtuin core domain and grouped into four classes
4	CCND1	Cyclin D1	The protein encoded by this gene belongs to the highly conserved cyclin family, whose members are characterized by a dramatic periodicity in protein abundance throughout the cell cycle. Cyclins function as regulators of CDK kinases
5	EZH2	Enhancer Of Zeste Homologue 2	This gene encodes a member of the polycomb‐group (PcG) family. PcG family members form multimeric protein complexes, which are involved in maintaining the transcriptional repressive state of genes over successive cell generations
6	CXCL8	C‐X‐C motif chemokine ligand 8	The protein encoded by this gene is a member of the CXC chemokine family and is a major mediator of the inflammatory response. The encoded protein is secreted primarily by neutrophils, where it serves as a chemotactic factor by guiding the neutrophils to the site of infection.
7	AR	Androgen receptor	The protein functions as a steroid‐hormone activated transcription factor. Upon binding the hormone ligand, the receptor dissociates from accessory proteins, translocates into the nucleus, dimerizes, and then stimulates transcription of androgen responsive genes
8	CDK4	Cyclin dependent kinase 4	The protein encoded by this gene is a member of the Ser/Thr protein kinase family. This protein is highly similar to the gene products of S. cerevisiae cdc28 and S. pombe cdc2. It is a catalytic subunit of the protein kinase complex that is important for cell cycle G1 phase progression

Abbreviation: CS‐DEG, cellular senescence‐differentially expressed genes.

### 3.5 MiRNA‐TF‐mRNA regulatory network analysis

**FIGURE 8:**
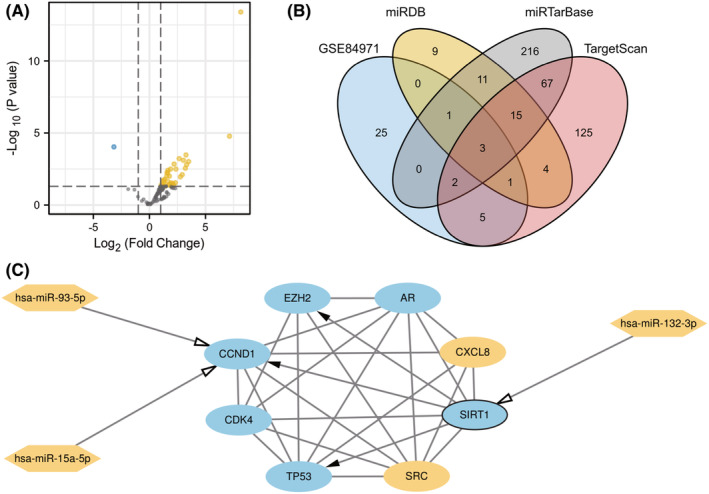
(A) The volcano map of GSE84971. (B) Venn diagram show that three overlapping miRNAs. (C) The miRNA‐TF‐mRNA regulatory network. Ellipse represent cellular senescence‐differentially expressed genes (CS‐DEGs), hexagon represent miRNAs and deepened black circle represent TF. Solid arrows indicate positive regulation and open arrows indicate negative regulation.

Based on the TRRUST database, we found that 3 CS‐DEGs act as TFs to regulate the expression of other genes, including TP53 (EZH2, TP53, CDK4, CCND1), SIRT1(CCND1, TP53, EZH2) and EZH2 (CCND1, TP53). Based on the GSE84971 dataset, we obtained 31 differentially expressed miRNAs (Figure 8A and Table S2). According to the miRWalk database, we predicted 127, 1070 and 928 upstream miRNAs from TargetScan, miRDB and miRTarBase for these 8 genes, respectively. After taking the intersection of Venn diagrams, we obtained 3 overlapping miRNAs, including hsa‐miR‐93‐5p (CCND1), hsa‐miR‐15a‐5p (CCND1), and hsa‐miR‐132‐3p (SIRT1) (Figure 8B). Finally, we constructed the miRNA‐TF‐mRNA regulatory network using Cytoscape (Figure 8C). Interestingly, SIRT1, as a TF, is regulated by the upstream hsa‐miR‐132‐3p and simultaneously regulates the expression of downstream CS‐DEGs (Figure 8C).

### 3.6 Results of RT‐qPCR analysis

**FIGURE 9:**
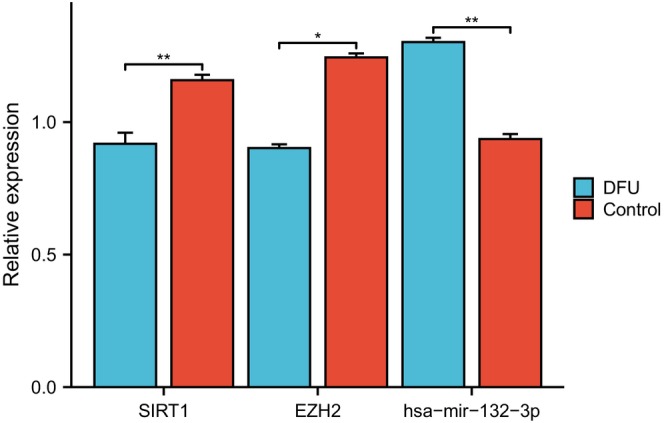
Relative expression of SIRT1, EZH2 and hsa‐mir‐132‐3p by RT‐qPCR analysis. *p < 0.05, **p < 0.01.

As shown in Figure 9, compared with the normal control group, the relative expression levels of SIRT1 and EZH2 in DFU samples are lower, while the expression level of hsa‐mir‐132‐3p is significantly higher. This is consistent with our previous analysis.

## 4 DISCUSSION

The diabetic environment, characterized by various forms of CS, leads to the secretion of SASP, which inhibits wound healing and results in systemic aging with serious pathogenic consequences.^5^, ^28^, ^29^, ^30^ Thus, CS is a pathogenic hub in the chronicity of diabetes‐associated wounds.^31^ This study identified DFU‐related and CS‐related genes from the GEO and CellAge databases. After Venn diagram calculation, 66 CS‐DEGs were obtained, including eight hub genes.

According to GSEA analysis, metabolic alterations and cell cycle arrest are hallmarks of CS.^32^ The KEGG pathway analysis revealed that the DEGs were mainly involved in CS, Kaposi sarcoma‐associated herpesvirus infection and Toll‐like receptor (TLR) signalling pathway in DFU. TLRs are one of the major sensors to regulate innate immunity.^33^ The findings suggest a strong interaction between P53 and TLR proteins, regulating various cellular processes such as DNA repair and replication, CS, differentiation, cell cycle arrest and tumour dynamics.^34^ Studies have shown that overexpression of TLR4 can induce CS in osteocytes, placental mesenchymal cells and dental pulp stem cells.^35^, ^36^, ^37^, ^38^ TLR2 and TLR5, drove CS of MSCs.^39^

We found eight overlapping hub genes, including TP53, SRC, SIRT1, CCND1, EZH2, CXCL8, AR and CDK4. P53 plays a crucial role in initiating CS by activating the P53/p21 pathway, which leads to cell cycle arrest.^40^ The tumour suppressor activity of P53 has been clarified, and recent studies have found that P53 has also been proved to be related to the pathogenesis of diabetes.^41^ Studies have revealed that P53 can contribute to insulin resistance and increase the risk of diabetes in mice. Knockout of P53 has been shown to ameliorate CS and reduce inflammation in adipose tissues of mice, which can help prevent the development of insulin resistance.^42^ Moreover, P53‐mediated senescence leads to reduced β‐cell self‐replication, massive islet depletion, and severe diabetes.^43^ Additionally, p21, as a downstream target of P53 and a regulator of the cell cycle, has been reported to play a role in the regulation of the regeneration capacity of pancreatic beta cells.^44^ Therefore, targeting P53 and p21 may hold promise for the treatment of diabetes. Recent studies have shown that inhibiting P53 expression can significantly promote the proliferation of human umbilical vein endothelial cells, reduce CS and significantly reduce cell cycle arrest. Hence, P53 and p21 are expected to be potential therapeutic targets for DFU.^45^

SIRT1 regulates various cellular biological processes, such as oxidative stress, apoptosis, inflammation and CS.^46^ The dysregulation of cellular processes that involve SIRT1 has been linked to the development of diabetes and its associated complications, highlighting the importance of SIRT1 as a potential therapeutic target for managing the disease.^47^ Accumulating findings have shown that SIRT1 inhibition leads to impaired angiogenesis, whereas SIRT1 overexpression promotes endothelial cell function and proliferation, playing a central role in angiogenesis.^48^ A recent study also showed that SIRT1 improves angiogenesis and wound healing in diabetic patients by reducing reactive oxygen species generation.^49^ Additionally, studies have shown that SIRT1 plays an important role in the healing process of chronic wounds in diabetic patients. Phellopterin cream has been shown to have anti‐inflammatory effects that facilitate healing of cutaneous wounds associated with diabetes through activation of SIRT1.^50^ Resveratrol advances the healing of diabetic wounds through the facilitation of angiogenesis via the SIRT1‐FOXO1‐c‐Myc signalling pathway.^51^ Similarly, nicotinamide riboside bolsters endothelial precursor cell functionality, thus accelerating the healing of refractory wounds by mediating the SIRT1/AMPK pathway.^52^ This reveals the potential of targeting SIRT1 modifiers as a therapeutic strategy to enhance the healing process of chronic diabetic wounds.

In diabetics, cellular DNA damage is prevalent and SIRT1 expression is diminished. In the bloodstream, hyperglycemia hinders the conversion of NADPH to NAD^+^, thereby restricting the availability of NAD^+^ substrates for SIRT1 and limiting its activation.^53^, ^54^ Consequently, the increased DNA breaks caused by hyperglycemia and the impaired repair due to reduced active MRN complexes make SIRT1 activation a promising target for DNA damage repair.^55^ Elevated glucose and free fatty acids stimulate endothelial progenitor CS through the PGC‐1α/SIRT1 signalling pathway.^56^ Capsaicin mitigates intermittent high glucose‐induced endothelial senescence via the TRPV1/SIRT1 pathway.^57^ As such, SIRT1 is critical in regulating the CS process for diabetic foot patients.

EZH2, the enzymatic catalytic subunit of the polycomb repressive complex 2, can modify the expression of downstream target genes through the trimethylation of Lys‐27 in histone 3 (H3K27me3). EZH2 can also regulate gene expression in other ways apart from H3K27me3. The roles of EZH2 in cell proliferation, apoptosis and senescence have been established.^58^ Likewise, EZH2 is crucial in the diabetic wound healing process. Research has demonstrated that the recruitment of EZH2‐mediated histone methylation and modulation of the HIF‐1α signalling pathway fosters fibroblast activation, consequently improving wound healing in DM.^59^ Findings have shown that EZH2 expression is down‐regulated in gestational diabetes mellitus (GDM), and overexpression of EZH2 promotes the proliferation and hinders the apoptosis of human umbilical vein endothelial cells.^60^

CCND1's most well‐known function is to form complexes with and activate CDK4/6. The CCND1‐CDK4/6 complex phosphorylates and inactivates the retinoblastoma (RB) protein, enabling the cell to transition from G1 to the S phase of the cell cycle.^61^ CCND1 is implicated in the cellular process of cell cycle arrest. SRC, a non‐receptor tyrosine kinase, is involved in various biological processes, including cell adhesion, cell cycle progression and cell migration.^62^ Additionally, SRC plays a role in the pathogenesis of CS in diabetes. Inhibition of SRC activation has been observed to reduce endogenous ROS production and increase ATP production in diabetic mice with hyperlipidemia.^63^ Safari‐Alighiarloo et al., identified SRC as a key gene for type 1 diabetes through gene expression profile analysis.^63^

We conducted an analysis of the miRNA‐TF‐mRNA regulatory network. Utilizing the TRRUST database, we identified three CS‐DEGs that function as TFs to modulate the expression of other genes, which include TP53, SIRT1 and EZH2. Concurrently, we predicted the upstream miRNA of CS‐DEGs. Ultimately, we established the miRNA‐TF‐mRNA regulatory network using Cytoscape software. Our findings revealed that SIRT1, acting as a TF, was regulated by the upstream hsa‐miR‐132‐3p, while also controlling the expression of downstream CS‐DEGs (CCND1, TP53 and EZH2).

SIRT1, a mammalian NAD^+^‐dependent sirtuin, has multifaceted functions in DNA repair, CS and cell metabolism through the deacetylation of various non‐histone proteins, including P53. Serving as a classical regulatory protein upstream of P53, SIRT1 enhances the degradation of P53 protein by interacting with P53 at the post‐transcriptional level. This interaction effectively modulates P53 and its downstream signalling pathways.^64^ Prior research has demonstrated that SIRT1 represses multiple TFs known to bind to the cyclin D1 gene promoter, such as NF‐kB, β‐catenin and AP‐1.^65^, ^66^, ^67^, ^68^, ^69^

The interaction between SIRT1 and EZH2 has been less discussed and only mentioned in a few cancer‐related diseases.^70^, ^71^, ^72^, ^73^ In liver fibrosis treatment research, when exposed to TGFβ1, SIRT1 activation substantially decreased EZH2 acetylation levels. However, EZH2 overexpression counteracted the SIRT1 activation that initially inhibited myofibroblast activity, suggesting that EZH2 is a target of SIRT1.^74^ Moreover, using an in vitro deacetylation assay, researchers verified that EZH2 is indeed a genuine substrate of SIRT1.^75^ Additionally, a previous study demonstrated that the depletion of SIRT1 increases the stability of EZH2 protein, and the level of EZH2 protein regulated by SIRT1 affects its repressive effects on target gene expression.^76^ However, the SIRT1/EZH2 axis has hardly been studied in the study of diabetic foot. In this paper, it has been summarized that SIRT1 and EZH2 both show regulatory effects in promoting CS and inhibiting wound healing under high glucose environment. Therefore, based on the findings of this study, we can speculate that upregulation of hsa‐mir‐132‐3p may lead to the downregulation of SIRT1 expression. As SIRT1 functions as a TF, its downregulation may lead to the downregulation of EZH2 expression, ultimately leading to CS. The accumulation of senescent cells may contribute to the long‐term nonhealing of DFU.

However, some limitations exist in this study. First, this is an analysis research based on sequencing data and public database. Second, although some previous studies have confirmed the role of these hub genes in regulating CS, they should be further verified in DFU model. Finally, the regulatory network we identified needs further functional experiments to verify.

## 5 CONCLUSIONS

In conclusion, the present study identified numerous hub genes and pathways that may be connected to the molecular mechanism underlying DFU. High glucose environment promotes CS through various molecular mechanisms, leading to poor healing of DFU. Eight hub genes were identified to regulate CS in DFU, including TP53, SRC, SIRT1, CCND1, EZH2, CXCL8, AR and CDK4. The hsa‐mir‐132‐3p/SIRT1/EZH2 axis is a potential target for the treatment of DFU.

## AUTHOR CONTRIBUTIONS

**Yike Huang:** Conceptualization (equal); data curation (equal); formal analysis (equal); writing – original draft (equal). **Dongqing Wang:** Data curation (equal); formal analysis (equal). **Wen Zhang:** Formal analysis (equal); supervision (equal). **Xue Yuan:** Project administration (equal). **Ke Li:** Investigation (equal); supervision (equal). **Yuanyuan Zhang:** Investigation (equal); supervision (equal). **Mingqiang Zeng:** Conceptualization (equal); investigation (equal); writing – review and editing (equal).

## CONFLICT OF INTEREST STATEMENT

The authors declare no conflict of interests.

## Supporting information


Table S1.

Table S2.

## Data Availability

The data analysed in this article comes from Gene Expression Omnibus (GEO) database (http://www.ncbi.nlm.nih.gov/geo). The accession number can be found in the materials and methods section of the article.
