# Supplementary material for: Identification of hub genes and pathways associated with cellular senescence in diabetic foot ulcers via comprehensive transcriptome analysis
Source: J Cell Mol Med. 2023 Nov 20;28(1):e18043. doi: 10.1111/jcmm.18043 (PMC10805497; doi:10.1111/jcmm.18043)
Supplement: Supplementary file 1 — Table S1. [file JCMM-28-e18043-s001.docx]

**Table s1.** The details of the overlapping CS-DEGs.

| **Gene** | **logFC** | **P.Value** |
| --- | --- | --- |
| PNPT1 | -1.24156 | 0.000306 |
| SENP7 | -1.31387 | 0.025395 |
| ENDOG | 0.7057 | 0.014703 |
| SORBS2 | -1.11045 | 0.001675 |
| MAGOHB | -1.92677 | 5.47E-05 |
| UBTD1 | 0.645942 | 0.023983 |
| IRF7 | 0.892277 | 0.010331 |
| NTN4 | -0.93445 | 0.000368 |
| TERF2 | -0.77253 | 0.001692 |
| XAF1 | -1.17577 | 0.019921 |
| BAG3 | 0.593053 | 0.007994 |
| CCND1 | -1.40636 | 2.99E-05 |
| CDK4 | -0.97889 | 0.001184 |
| BCL6 | -1.49731 | 0.000323 |
| BLK | 0.546757 | 0.012093 |
| WWP1 | -1.14068 | 0.007716 |
| IGFBP5 | -1.38363 | 0.011941 |
| KDM4A | -0.94817 | 0.032882 |
| AXL | -0.77569 | 0.011153 |
| VENTX | 0.955823 | 0.002974 |
| MVK | 0.663928 | 0.019477 |
| TERT | 0.672826 | 0.012255 |
| SOCS1 | 1.238785 | 0.013319 |
| PDZD2 | -1.96253 | 6.04E-06 |
| SRC | 0.699404 | 0.038863 |
| TP53 | -1.50085 | 0.003067 |
| BMI1 | -0.83018 | 0.018783 |
| SIRT1 | -0.71739 | 0.033318 |
| ASF1A | -1.18516 | 0.00257 |
| DEK | -1.07237 | 0.044658 |
| RSL1D1 | -2.40018 | 0.003115 |
| LIMK1 | 0.733809 | 0.005578 |
| MAP2K3 | 1.128668 | 0.016446 |
| AAK1 | -0.86386 | 0.034552 |
| HMGB1 | -1.69136 | 1.06E-06 |
| HIVEP1 | -1.08272 | 0.012887 |
| DHCR24 | -1.02554 | 0.020306 |
| MATK | 0.768938 | 0.005729 |
| MECP2 | -0.75859 | 0.001547 |
| NEK4 | -0.93132 | 0.003313 |
| ZNF148 | -1.32138 | 0.001968 |
| EZH2 | -1.0284 | 0.0188 |
| CXCL8 | 3.528432 | 0.002017 |
| PBRM1 | -1.17132 | 0.014341 |
| PIM1 | 0.789955 | 0.030617 |
| MAP2K6 | -1.38939 | 0.000167 |
| ASPH | -1.25622 | 0.012549 |
| MAPK14 | -0.52904 | 0.042633 |
| CBX8 | 0.734939 | 0.00414 |
| SFN | 1.169792 | 0.001684 |
| SPIN1 | -1.47391 | 0.000165 |
| TXNIP | -1.86987 | 0.001747 |
| TLR3 | -2.023 | 7.44E-06 |
| ATF7IP | -2.08864 | 0.000431 |
| EPHA3 | -0.69127 | 0.025488 |
| MAPKAPK5 | -1.15189 | 0.000254 |
| OTX2 | 0.87202 | 0.003459 |
| SP1 | -1.1032 | 2.74E-05 |
| IGFBP1 | 0.733388 | 0.004032 |
| IRF5 | 0.606808 | 0.009467 |
| KSR2 | 0.675074 | 0.013887 |
| MAP2K1 | 0.71194 | 0.009663 |
| TXN | 1.745253 | 2.79E-05 |
| AR | -1.24837 | 0.002144 |
| TP63 | -0.85131 | 0.019605 |
| GATA4 | 0.804167 | 0.003237 |
