# Supplementary material for: Identification of hub genes and pathways associated with cellular senescence in diabetic foot ulcers via comprehensive transcriptome analysis
Source: J Cell Mol Med. 2023 Nov 20;28(1):e18043. doi: 10.1111/jcmm.18043 (PMC10805497; doi:10.1111/jcmm.18043)
Supplement: Supplementary file 2 — Table S2. [file JCMM-28-e18043-s002.docx]

**Table s2.** The details of the differentially expressed miRNAs.

| **ID** | **logFC** | **P.Value** |
| --- | --- | --- |
| hsa-miR-143-3p | 8.1451 | 4.01E-14 |
| hsa-miR-21-5p | 7.1353 | 1.67E-05 |
| hsa-miR-302d-3p | -3.1688 | 9.20E-05 |
| hsa-miR-181a-5p | 3.2546 | 3.35E-04 |
| hsa-miR-487b | 2.6541 | 5.96E-04 |
| hsa-miR-324-5p | 3.0247 | 7.86E-04 |
| hsa-miR-132-3p | 3.4898 | 9.57E-04 |
| hsa-miR-379-5p | 2.198 | 1.44E-03 |
| hsa-miR-450a-5p | 3.3123 | 1.59E-03 |
| hsa-miR-99a-5p | 3.1986 | 2.79E-03 |
| hsa-miR-374a-5p | 1.8839 | 3.18E-03 |
| hsa-miR-24-3p | 2.3632 | 3.22E-03 |
| hsa-miR-145-5p | 1.6463 | 3.90E-03 |
| hsa-miR-98 | 1.5675 | 5.53E-03 |
| hsa-miR-34a-5p | 1.6253 | 6.02E-03 |
| hsa-miR-93-5p | 2.9213 | 7.80E-03 |
| hsa-miR-543 | 1.5449 | 9.63E-03 |
| hsa-miR-574-3p | 1.7749 | 9.95E-03 |
| hsa-miR-140-5p | 2.7554 | 1.11E-02 |
| hsa-miR-23b-3p | 1.3473 | 1.62E-02 |
| hsa-miR-214-3p | 1.2955 | 1.98E-02 |
| hsa-let-7d-5p | 1.1968 | 2.19E-02 |
| hsa-miR-376a-3p | 1.6409 | 2.35E-02 |
| hsa-miR-15a-5p | 1.2729 | 2.66E-02 |
| hsa-miR-4286 | 2.3055 | 2.86E-02 |
| hsa-miR-196b-5p | 1.9322 | 2.93E-02 |
| hsa-miR-146a-5p | 2.0725 | 3.20E-02 |
| hsa-miR-494 | 1.9998 | 3.33E-02 |
| hsa-miR-152 | 1.7808 | 3.86E-02 |
| hsa-miR-185-5p | 1.7448 | 3.97E-02 |
| hsa-let-7i-5p | 1.0529 | 4.06E-02 |
| hsa-miR-107 | 2.1558 | 4.20E-02 |
| hsa-miR-199a-5p | 1.1943 | 4.26E-02 |
| hsa-miR-27b-3p | 1.1167 | 4.71E-02 |
| hsa-miR-193a-5p | 1.0859 | 4.84E-02 |
| hsa-miR-485-3p | 1.9734 | 4.84E-02 |
| hsa-miR-148a-3p | 1.3803 | 4.95E-02 |
